# Supplementary material for: Cryo-EM structure of photosystem II supercomplex from a green microalga with extreme phototolerance
Source: Nat Commun. 2026 Jan 9;17:341. doi: 10.1038/s41467-025-65861-2 (PMC12789144; doi:10.1038/s41467-025-65861-2)
Supplement: Supplementary file 1 — Supplementary Information [file 41467_2025_65861_MOESM1_ESM.pdf]

Supplementary Information

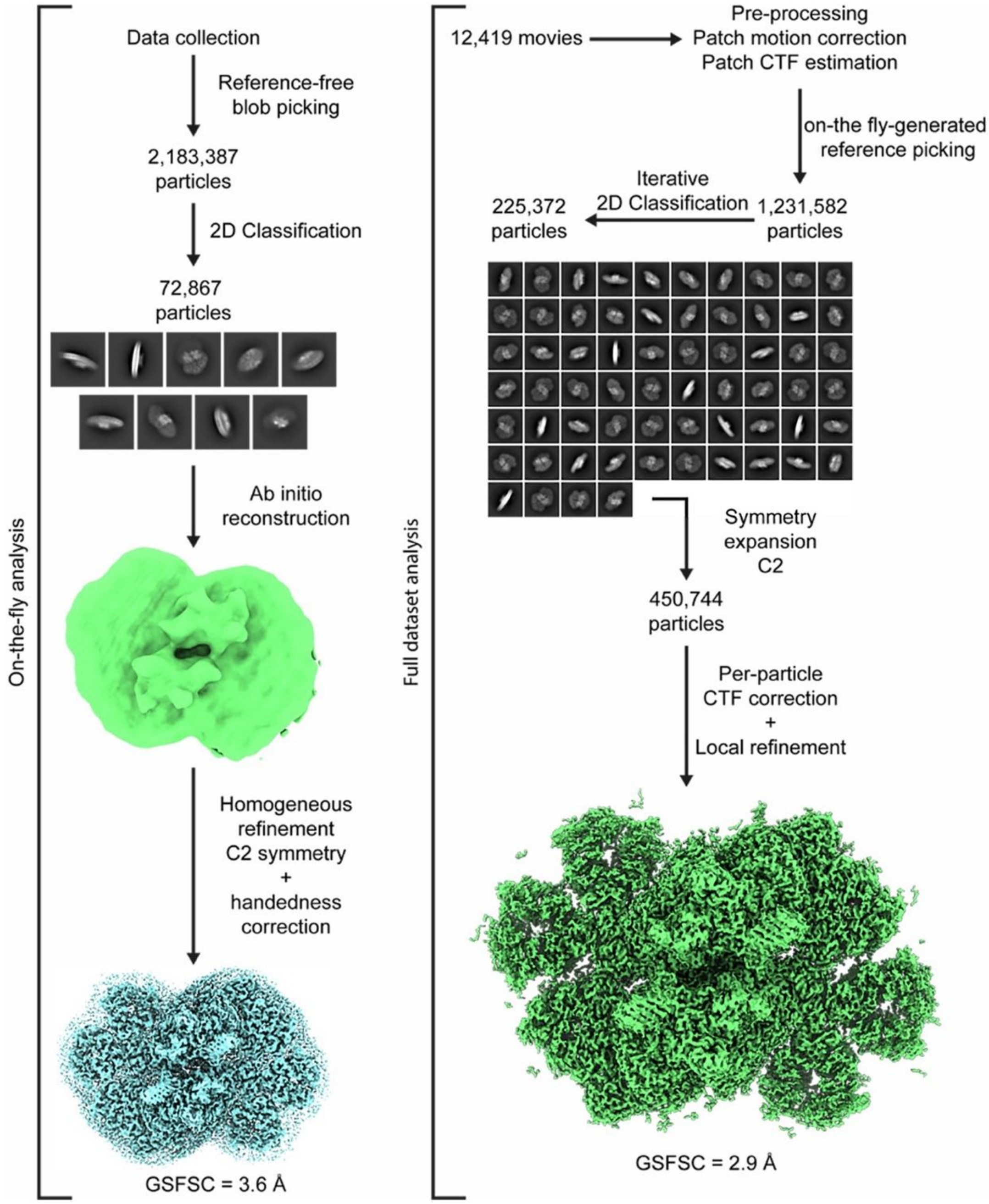

Supplementary Fig. 1 | The workflow of cryo-EM data processing and reconstruction of 3D map of PSII-LHCII supercomplex. The EM-map of the $C_2S_2M_2L_2$ PSII-LHCII supercomplex was derived in two distinct steps: on the left, the workflow applied on-the-fly, parallel to data collection, is visible, with all parameters pre-set, leading to a reconstruction of 3.6 Å (FSC = 0.143). On the right, the manual analysis workflow can be seen, which resulted in a final reconstruction of 2.9 Å (FSC = 0.143), leveraging the initial reconstruction derived from the on-the-fly analysis.

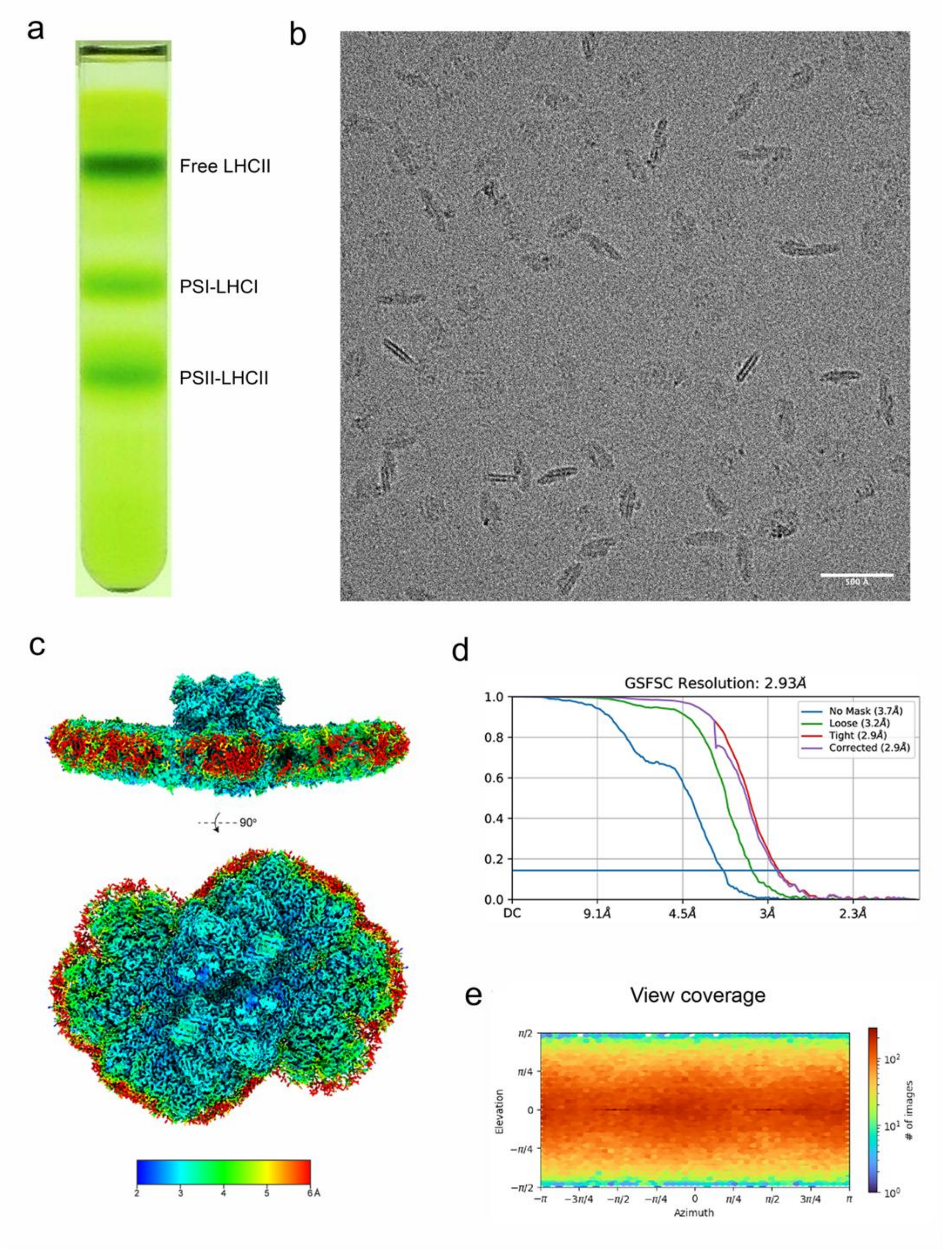

Supplementary Fig. 2 | Separation of *C. ohadii* thylakoid membrane complexes and evaluation of cryo-EM maps. a, Saperation of thylakoid membrane proteins by the method of sucrose gradient ultracentrifugation. The bands representing PSII-LHCII, PSI-LHCI, and free LHCII are indicated. **b**, A representative electron micrograph of cryo-EM specimen from *C. ohadii* PSII-LHCII supercomplex. c-e, FSC curve, and local resolution estimation map for the final complex reconstruction. **c**, Local resolution estimation of the $C_2S_2M_2L_2$ supercomplex map, ranging from 2 to 6 Å. **d**, Gold-standard (0.143) Fourier Shell Correlation curve for $C_2S_2M_2L_2$ complex at 2.93 Å resolution. **e**, View coverage distribution of the particles used in the final map reconstruction, displaying a fairly uniform particle distribution.

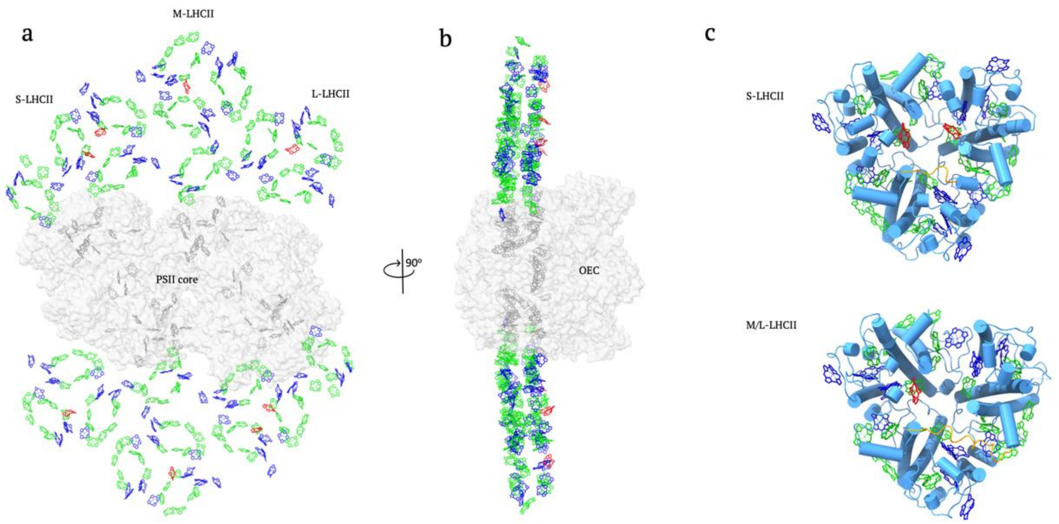

Supplementary Fig. 3 | Arrangement and positions of chlorophylls in PSII $C_2S_2M_2L_2$ supercomplex in *C. ohadii*. **a**, A top view of PSII-LHCII supercomplex from the lumenal side showing the arrangement of chlorophylls. **b**, A side view of PSII-LHCII along the membrane plane showing the lumenal and stromal layers of chlorophylls. Chlorophyll *a* and *b* molecules are indicated in green and blue, respectively. The new chlorophyll sites (Chl *a*615) are shown in red. The core complex is shown in surface representation, while chlorophylls are represented in stick models. **c**, A detailed view of the protein-pigments assembly of LHCII antenna in *C. ohadii*. The helices are shown in the tubular representation, the chlorophylls are in the stick model. The new chlorophylls (in red) are coordinated by two chains in S-LHCII, whereas the second Chl *a*615 site could not be resolved in M- and L-LHCII due to the limited map resolution. Note the presence of a long loop region (in orange) that can hinder the assembly of new chlorophyll in the corresponding chains of S-/M- and L-LHCII.

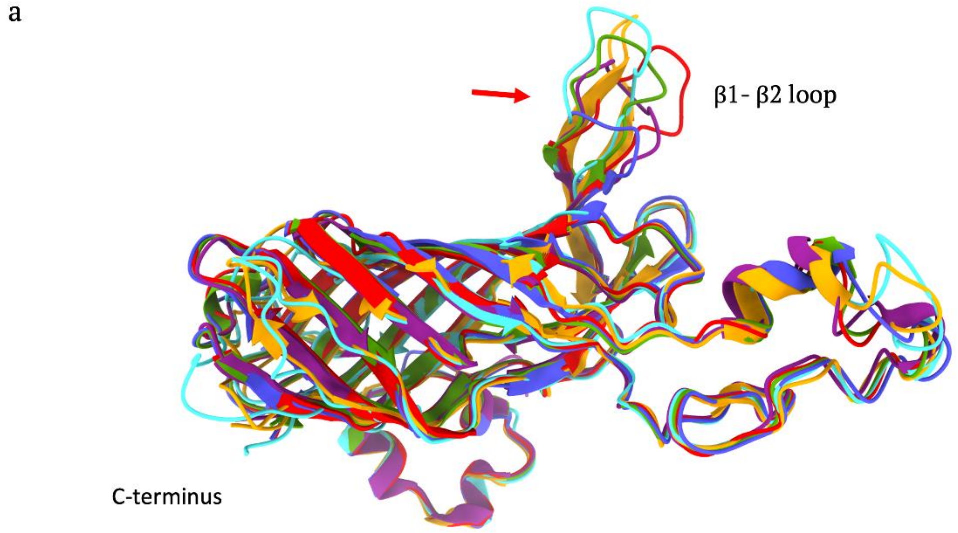

b

```
PsbO_C. ohadii  22  ANQCPILGADAQQGLGDVKPGTFQLDKFCMEPSSFKVKMP------GSKEFEPSTLLTRNTYSLDQMTGS  85
PsbO_8BD3       20  ..T..T.-DSGSSNVK.L....YK.G......T..T..EE-SQFKG.DA..V.TK.M..L..T..E.S.Q  87
PsbO_6KAC       71  ..T..V.-ESGTTN.KEL.A.SYK.EN..I..T..T..EE-SQFKG.ET..VKTK.M..L..T..A.S..  138
PsbO_6JLU       21  ..R.AEV-VGED-SIKPKPG--AK.VDM.I..KAWA.EEEVGKAGKTE.K.VN.KVM..Q..T..AIE.P  86
PsbO_7Y5E       53  ..R..EV-DGTKGSIKIE.GKKYKIVDM.I..K..Q.EDEVGKGKQAR...VDTK.M..A..T.TGIE.P  121
PsbO_5XNL       26  .....TI-DGGVDSFSFKPGK-YNAK.L.L..T..T..SE-GVTKNTPLA.QNTK.M..L..T..EIE.P  92
```

Supplementary Fig. 4 |Comparison of structural models and amino acid sequences of PsbO protein in different organisms. a) Overlay of the structural models of PsbO subunit from *C. ohadii* on the PsbO models from previous *C. ohadii* structure (8BD3), *C. reinhardtii* (6KAC), *C. gracilis* (6JLU), *P. purpureum* (7Y5E) and *P. sativum* (5XNL). All the protein domains are similar except for the presence of a shorter β1-β2 loop in our *C. ohadii* structure. PsbO protein from our structure, previous *C. ohadii*, *C. reinhardtii, C. gracilis, P. purpureum*, *P. sativum* are shown in blue, green, red, orange, cyan, and magenta, respectively. **b)** Alignment of sequences of PsbO protein from different organisms. The missing residues of β1-β2 loop in *C. ohadii* are shown in a red rectangle. Amino acids identical to those in the reference sequence are indicated by dots. A dash denotes a gap introduced to optimize the alignment, corresponding to the absence of an amino acid at that position.

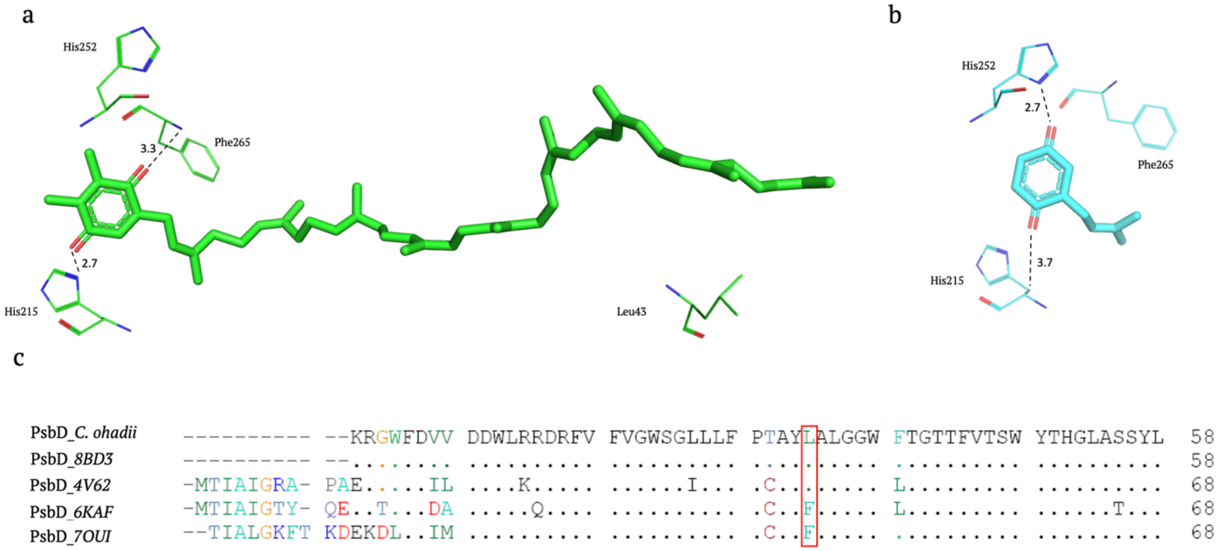

Supplementary Fig. 5 |Specific interactions of plastoquinone molecule at Q_B site in *C. ohadii* and *C. reinhardtii*. **a)** H-bond interactions of D1-His215 and D1-Phe265 with the head part of plastoquinone molecule at Q_B site in *C. ohadii*. D2-Leu43 interacts with the tail of plastoquinone through hydrophobic interaction. **b)** H-bond interactions of D1-His215 and D1-His252 with the head part of plastoquinone molecule at Q_B site in *C. reinhardtii*. **c)** Comparison of N-terminal amino acid sequences of PsbD (D2) protein in *C. ohadii* (8BD3), cyanobacteria (4V62), *C. reinhardtii* (6KAF), and Arabidopsis (7OUI). D2-Leu43 involved in the interaction with the tail of the plastoquinone molecule is shown in a red rectangle. Amino acids identical to those in the reference sequence are indicated by dots. A dash denotes a gap introduced to optimize the alignment, corresponding to the absence of an amino acid at that position.

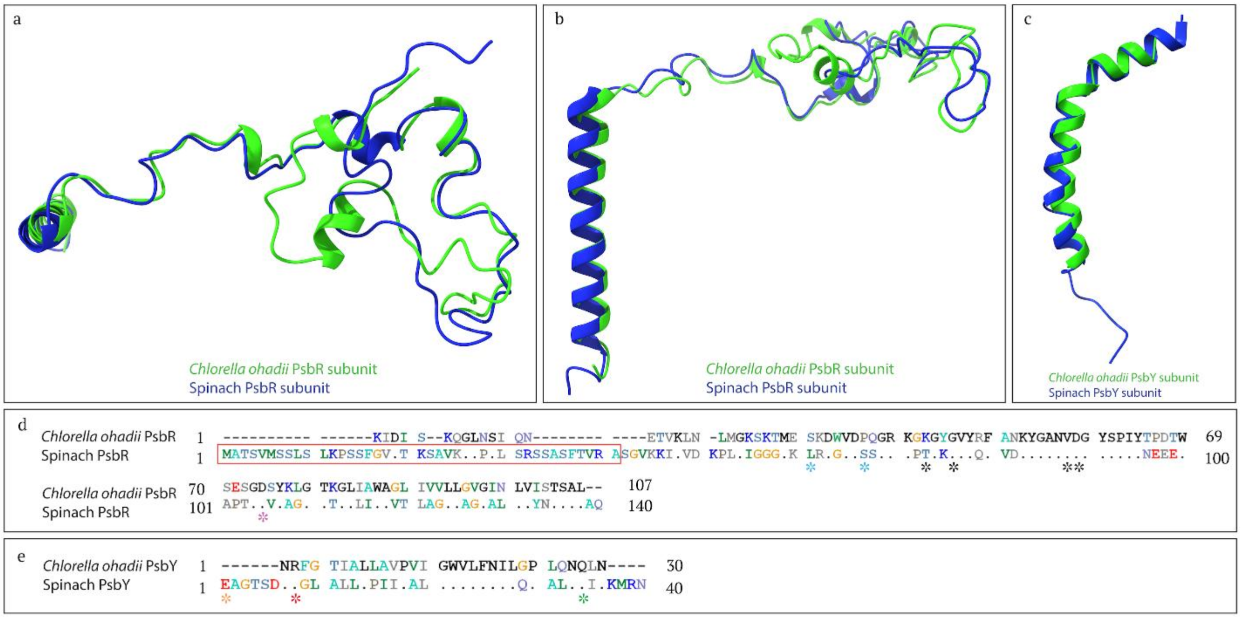

Supplementary Fig. 6| Comparison of structural models and amino acid sequences of PsbR and PsbY subunits in *C. ohadii* and spinach (PDB 8Z9D). a-c) superposition of structural models of PsbR and PsbY subunits in *C. ohadii* (green) and spinach (blue). **d-e)** alignment of amino acid sequences of PsbR and PsbY proteins. The residues of PsbR and PsbY subunits interacting with the core proteins PsbC, D, E, F, J and P are highlighted with cyan, black, red, green, magenta and orange asterisks. The unmodelled residues of the spinach PsbR subunit are in a red rectangle. Amino acids identical to those in the reference sequence are indicated by dots. A dash denotes a gap introduced to optimize the alignment, corresponding to the absence of an amino acid at that position.

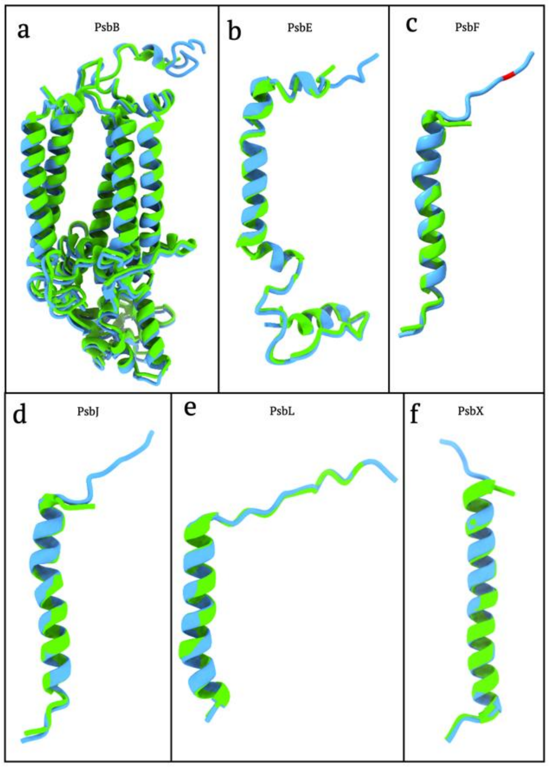

Supplementary Fig. 7 | Superposition of models of the PsbB, E, F, J, L, and X subunits in *C. ohadii* on the structural models of the corresponding subunits in *C. reinhardtii* (6KAC). a-f, The superpositions of the structural models of the specific Psb subunits from *C. ohadii* and *C. reinhardtii* show the presence of regions with longer loops in PsbB, E, F, J, L, and X subunits. The corresponding helices from *C. ohadii* and *C. reinhardtii* are shown in blue and green, respectively. The N-terminal Tyr7 residue, which is replaced in *C. ohadii*, is indicated in red.

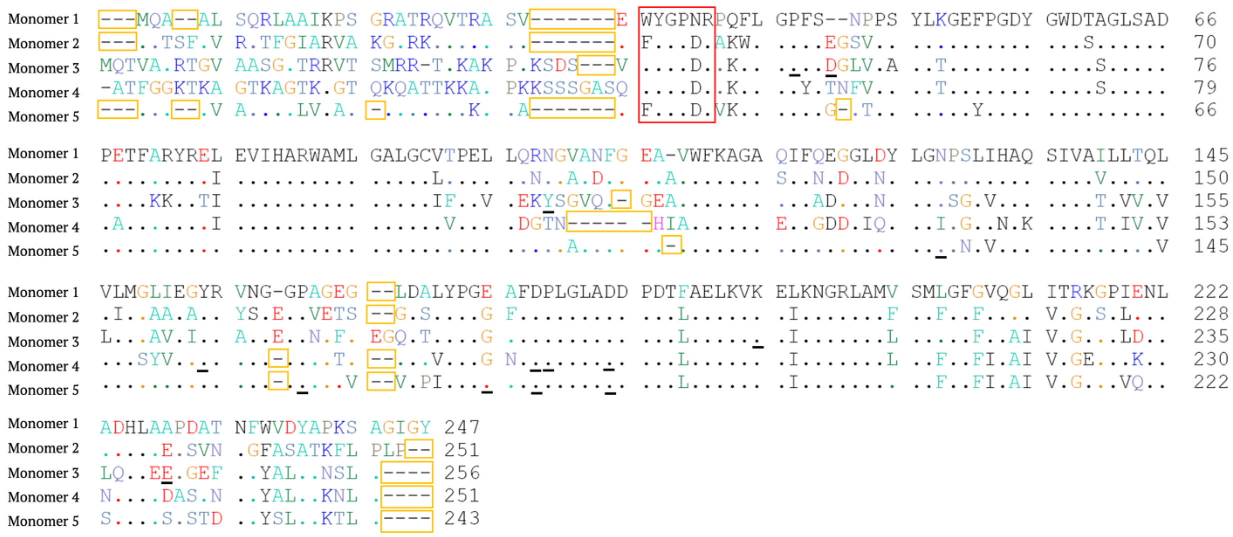

Supplementary Fig. 8 | Sequence alignment of light-harvesting LHCII antenna proteins of PSII-LHCII supercomplex from *C. ohadii.* The listed amino acid sequences were used to model the monomers 1-5 of LHCII antenna in *C. ohadii*. The non-conserved residues are colored and the conserved residues are indicated as a dot. The trimerization motifs of the Lhcb monomers are shown in a red rectangle. The missing residues in the sequences are indicated the in orabnge rectangle. The residues of monomers 3, 4, and 5 (Fig. 3) involved in the interactions with the PSII core and antenna are underlined. The interacting residues refer to the Supplementary Table 4. Amino acids identical to those in the reference sequence are indicated by dots. A dash denotes a gap introduced to optimize the alignment, corresponding to the absence of an amino acid at that position.

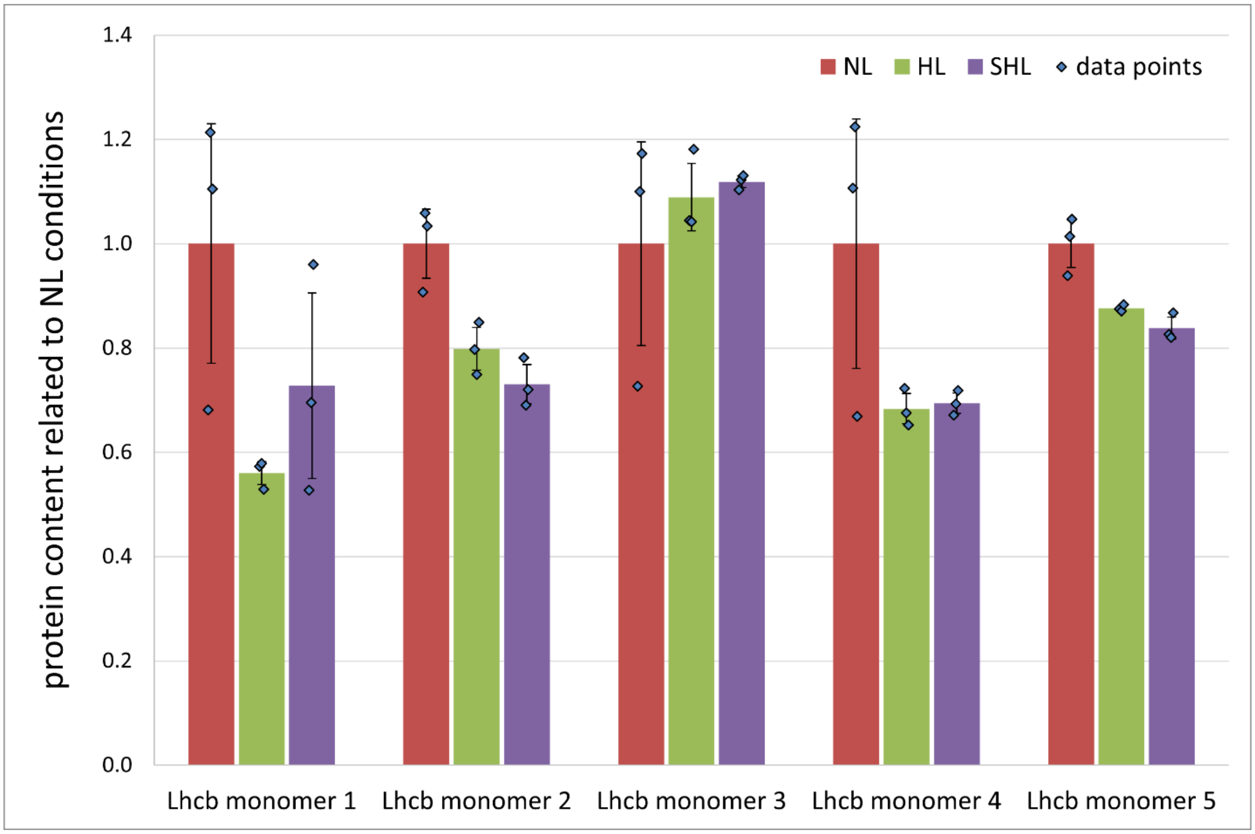

Supplementary Fig. 9 | Relative content of light-harvesting proteins in thylakoid membranes of *C. ohadii* grown under different light intensities. The content of individual light-harvesting proteins (monomer 1-5) was evaluated relative to the content of the D1 protein of PSII and subsequently normalized to cells grown under NL conditions. The protein content was determined in isolated thylakoid membranes by LC-MS/MS. The presented values are means ± SD from three replicates (n=3). Light intensities were 100 µmol photons m^{-2} s^{-1} (NL), 1700 µmol photons m^{-2} s^{-1} (HL), and 2500 µmol photons m^{-2} s^{-1} (SHL). Source data are provided as a Source Data file.

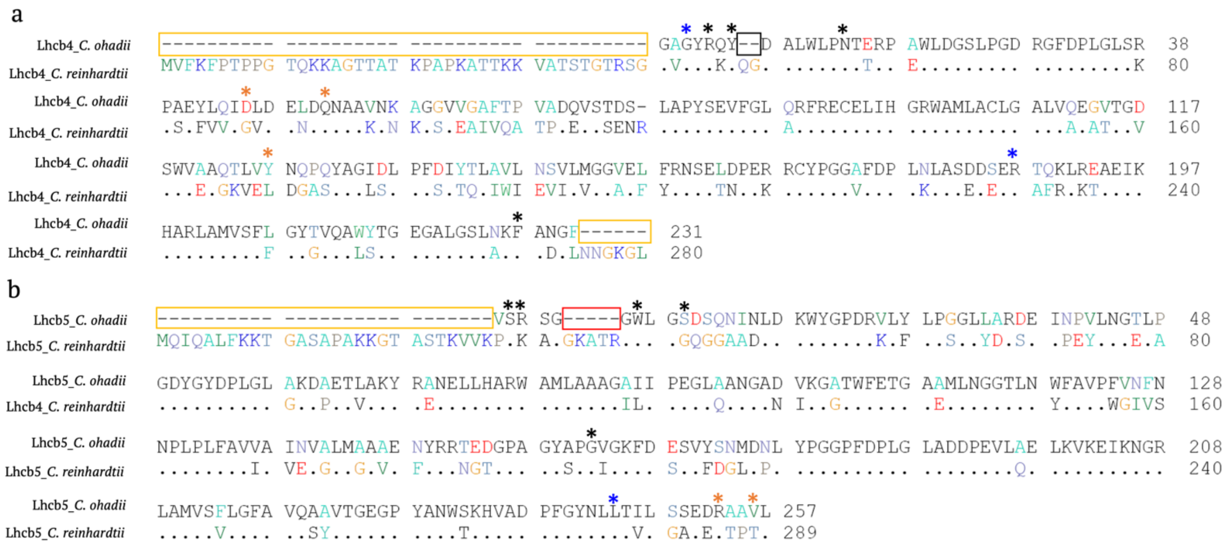

Supplementary Fig. 10 | A comparison of the sequences of light-harvesting monomers Lhcb4 and Lhcb5 between *C. ohadii* and *C. reinhardtii.* **a**, the alignment of the sequence of *C. ohadii* Lhcb4 protein with its counterpart from *C. reinhardtii.* The *C. ohadii* residues interacting with PsbB, L-LHCII, and M-LHCII are indicated by orange, black, and blue asterisks, respectively. **b**, the alignment of the sequence of *C. ohadii* Lhcb5 protein with its counterpart from *C. reinhardtii.* The *C. ohadii* Lhcb5 residues interacting with PsbC (CP43), S-LHCII, and PsbZ are indicated by orange, black, and blue asterisks, respectively. The missing N and C-terminal residues are highlighted in orange rectangles. The N-terminal residues of Lhcb4 that are missing in *C. ohadii* structure are indicated in the black rectangle. The deletion of amino acid residues of *C. ohadii* Lhcb5 protein is indicated in the red rectangle. Amino acids identical to those in the reference sequence are indicated by dots. A dash denotes a gap introduced to optimize the alignment, corresponding to the absence of an amino acid at that position.

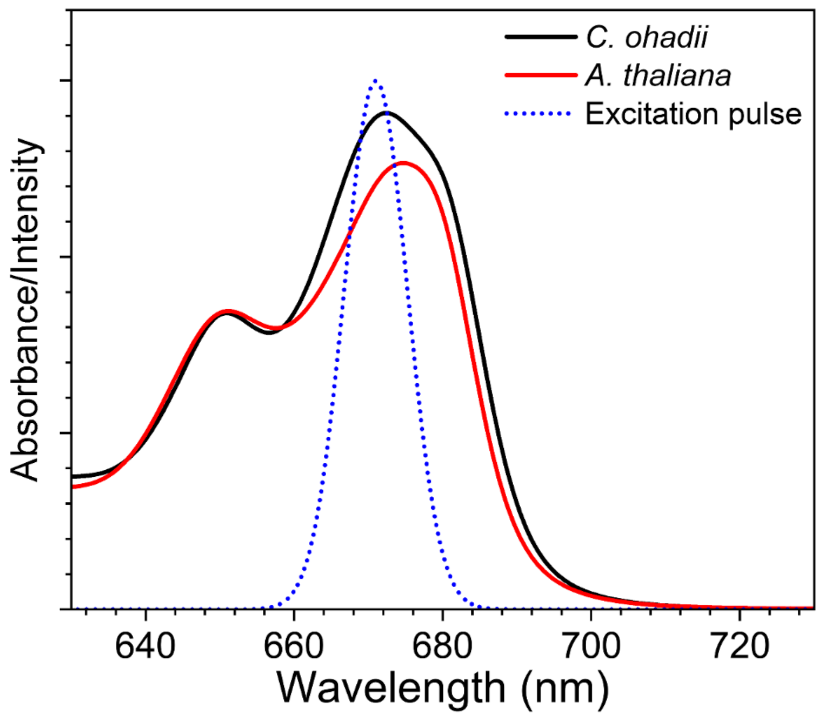

Supplementary Fig. 11| Overlap of the pump spectrum used in the transient absorption experiments and the absorption spectrum of the LHCII of *C. ohadii* and *A. thaliana* in the Q_y region. Source data are provided as a Source Data file.

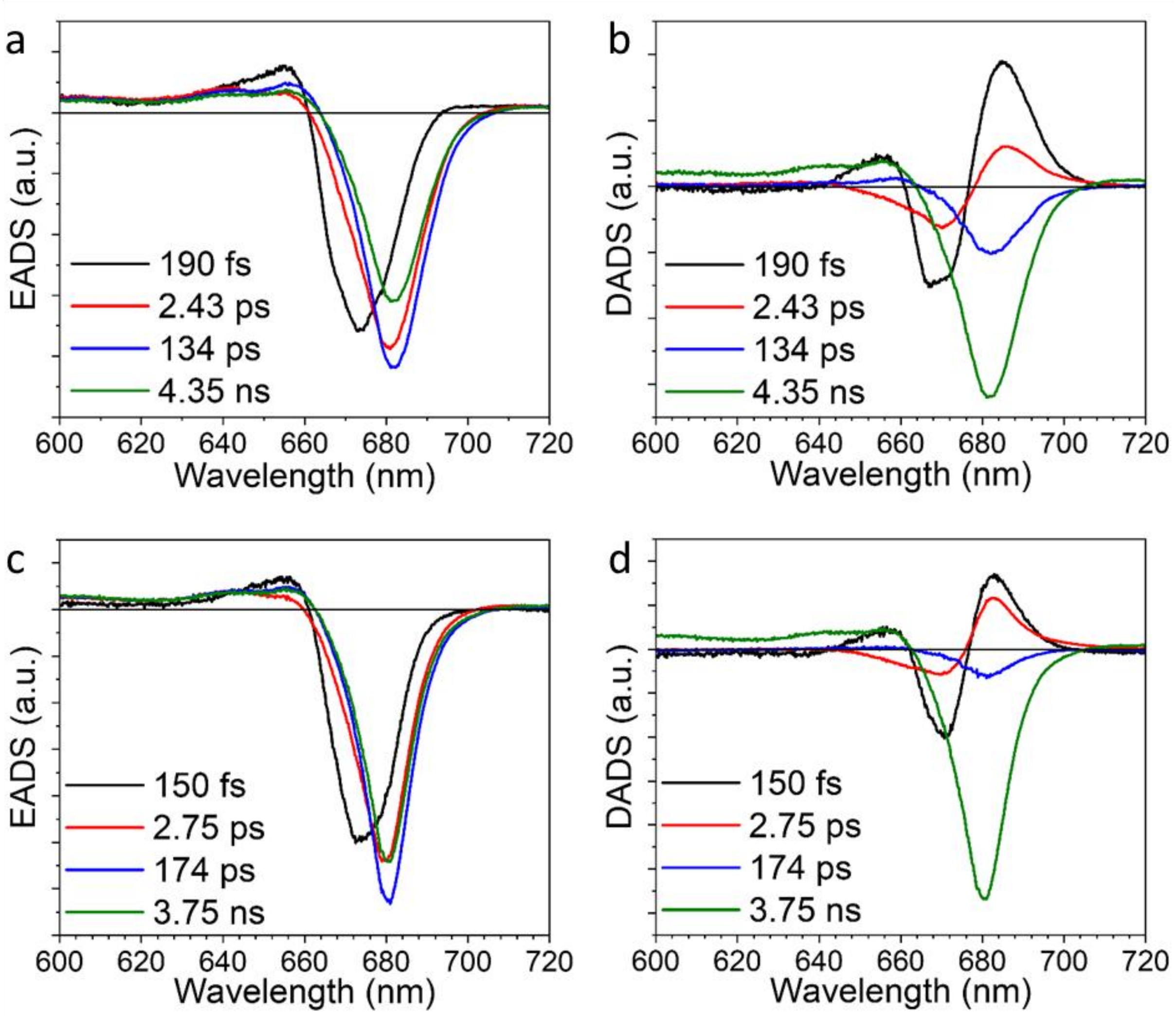

Supplementary Fig. 12 | Full set of EADS (a, c) and DADS (b, d) that were obtained from the global analyses on the transient absorption data of the LHCII trimers excited at 671 nm. a, **b** LHCII trimer of *C. ohadii* and **c, d** of *A. thaliana*. Source data are provided as a Source Data file.

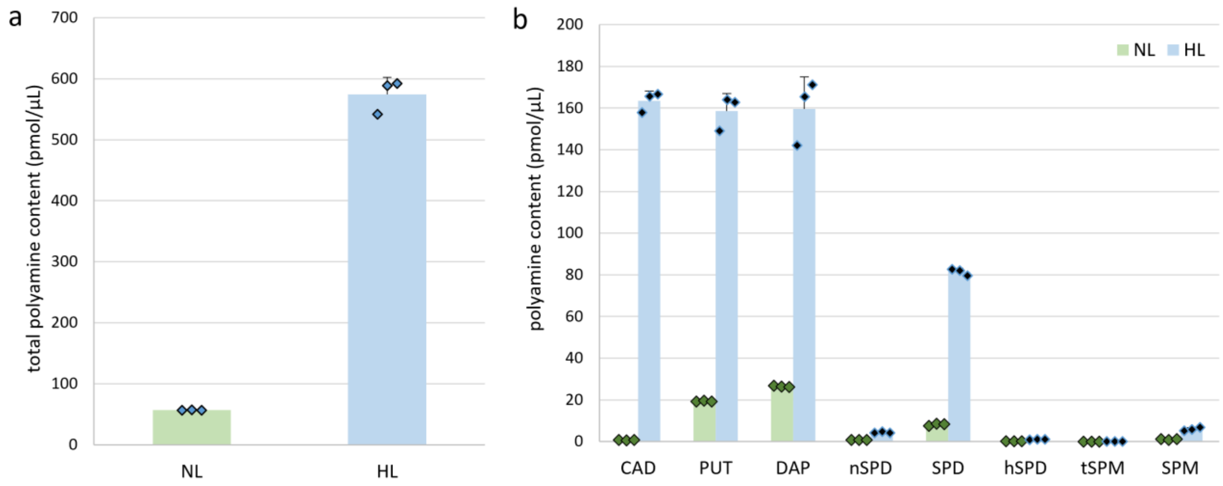

Supplementary Fig. 13 | Polyamine content in thylakoid membranes of *C. ohadii* grown under 100 µmol photons m^{-2} s^{-1} (NL) and 1700 µmol photons m^{-2} s^{-1} (HL). a, Total content of polyamines and **b,** the content of individual polyamines in thylakoid membranes of NL and HL *C. ohadii*. The presented values are means ± SD from three replicates (n=3). Abbreviations used: Cadaverine (CAD), Putrescine (PUT), Diaminopropane (DAP), Norspermidine (nSPD), Spermidine (SPD), Homospermidine (hSPD), Thermospermine (tSPM), Spermine (SPM). Source data are provided as a Source Data file.

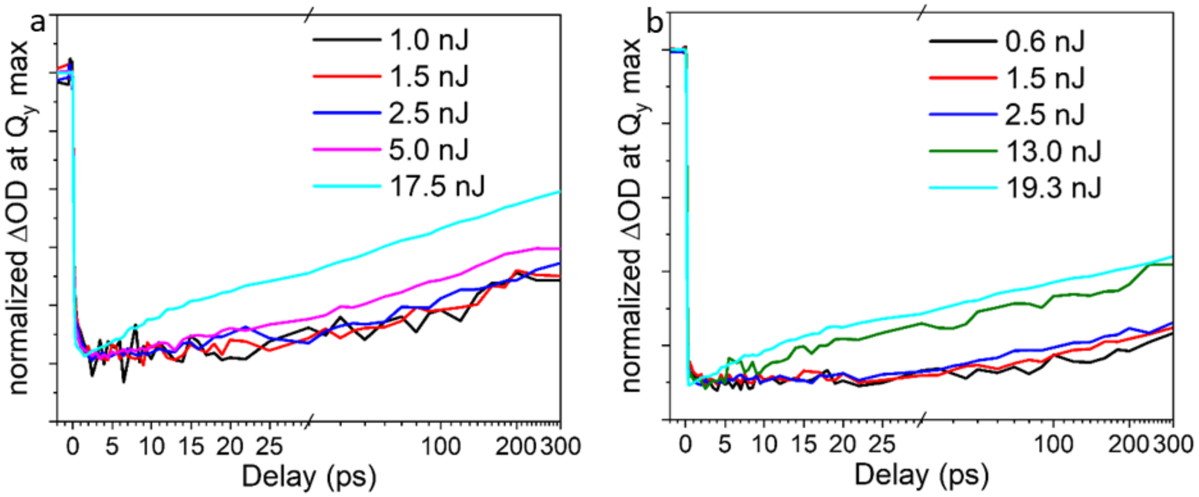

Supplementary Fig. 14 | Power studies for the transient absorption experiments on the LHCII of *C. ohadii* (a) and *A. thaliana* (b). For the measurements that are presented in the manuscript a power of 1.5 nJ was chosen for the *C. ohadii* LHCII and 0.6 nJ for that of *A. thaliana*. Source data are provided as a Source Data file.

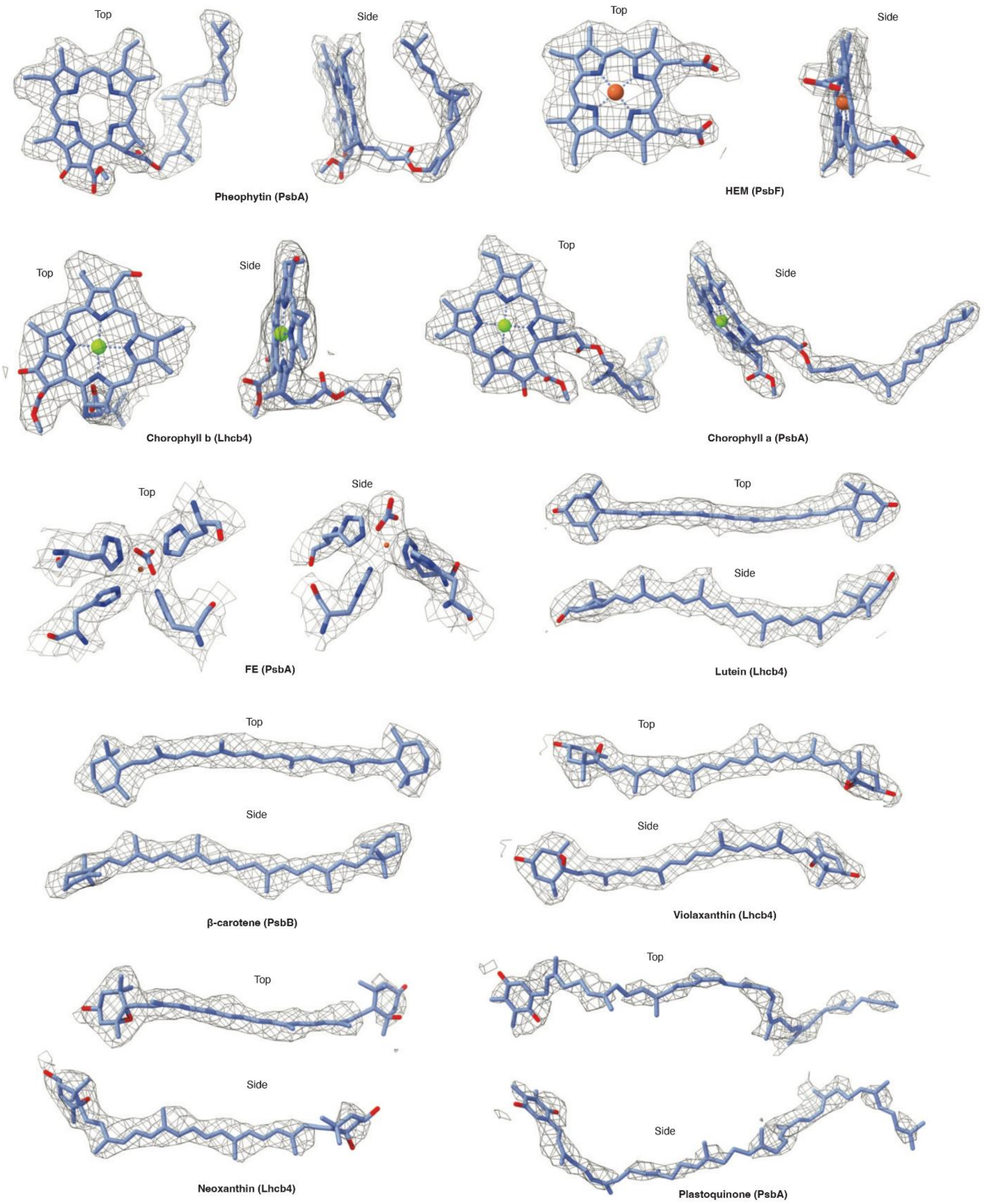
Top
Side
Pheophytin (PsbA)
Top
Side
HEM (PsbF)
Top
Side
Chorophyll b (Lhcb4)
Top
Side
Chorophyll a (PsbA)
Top
Side
FE (PsbA)
Top
Side
Lutein (Lhcb4)
Top
Side
β-carotene (PsbB)
Top
Side
Violaxanthin (Lhcb4)
Top
Side
Neoxanthin (Lhcb4)
Top
Side
Plastoquinone (PsbA)

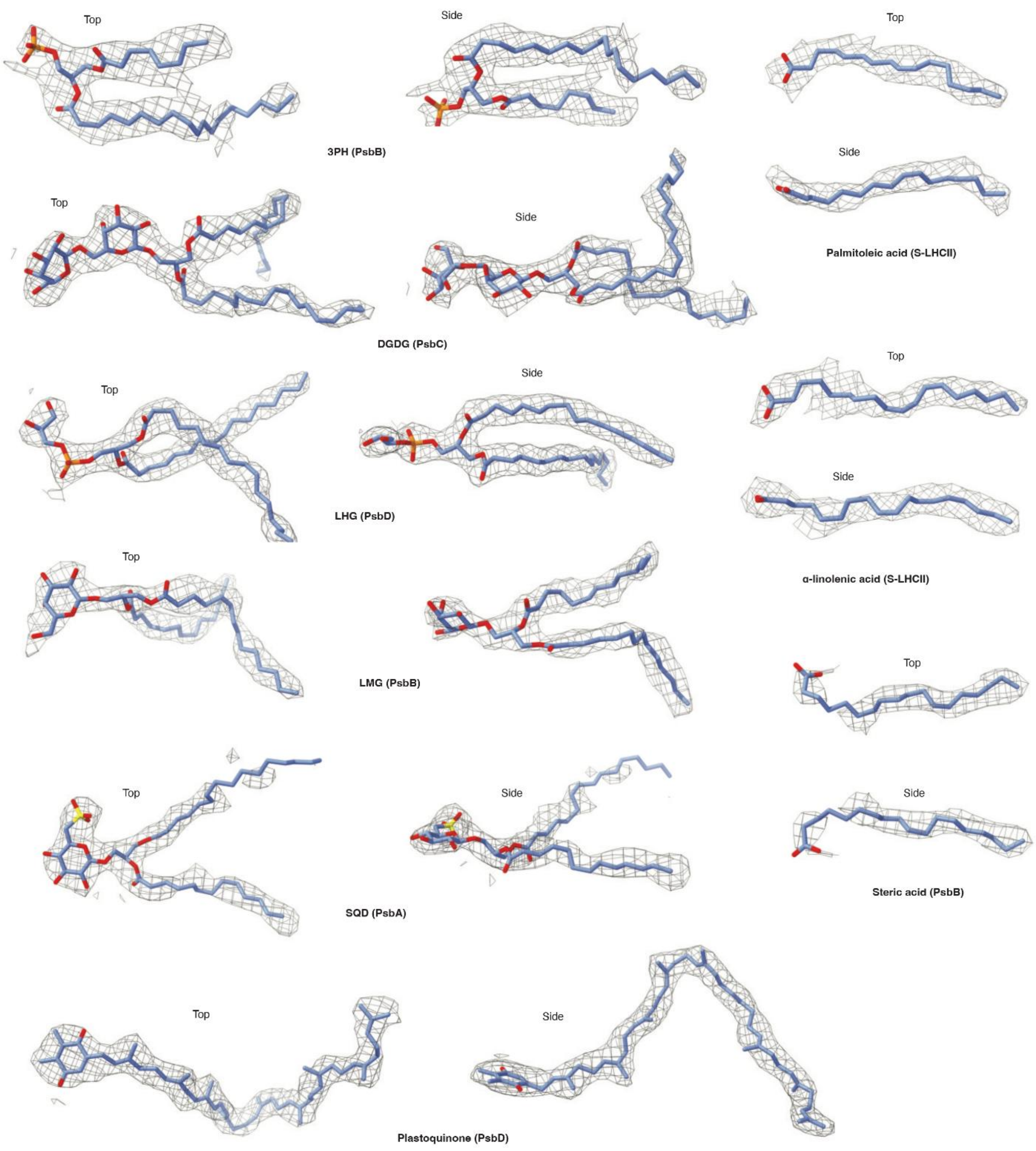

Supplementary Fig. 15 | Representative cryo-EM densities of various ligands bound to the PSII $C_2S_2M_2L_2$ supercomplex. The ligands are shown as sticks. Fe and Mg are shown as orange and green spheres. Carbon backbone of ligands is depicted in light blue, nitrogen and oxygen atoms are shown in blue and red.

Supplementary Table 1 | Cryo-EM data collection, refinement, and validation statistics for the structural model of $C_2S_2M_2L_2$ supercomplex from *C. ohadii.*

**Data collection**	
Magnification	150 000 x
Voltage (kV)	200
Focal length/Cs (mm)	3.4/2.7
Objective aperture (µm)	100
Number of movies collected	12,419
Electron exposure (e^{-}/Å^2)	90
Defocus range (µm)	-0.5 to -3.0
Pixel size (Å)	0.948
**Data Processing**	
Symmetry imposed	C2
Initial particle images (no.)	1,231,582
Final particle images (no.)	450,744
Map resolution (Å)/FSC threshold	2.93/0.143
Map sharpening B-factor (Å^2)	-83.8
**Refinement**	
Resolution (Å)	2.93
Mask CC	0.821
Volume CC	0.791
Initial model (PDB)	8C29 and 7OUI
**Model composition**	
Non-hydrogen atoms	112944
Protein residues	10 624
Ligands	614

**Average B factors (Å^2)**	
Overall	55.3
Protein	55.9
Ligands	53.7
**R.M.S. deviations**	
Bond lengths (Å)	0.008
Bond angles (°)	1.117
**Validation**	
MolProbity score	1.85
Clash score	7.87
Rotamer outliers (%)	2.28
**Ramachandran plot**	
Favored (%)	97.19
Allowed (%)	2.81
Disallowed (%)	0.00

Supplementary Table 2 | List of cofactors modelled in *C. ohadii* $C_2S_2M_2L_2$ supercomplex.

Abbreviations: DGDG, digalactosyldiacyl glycerol; LHG, dipalmitoyl phosphatidyl glycerole; LMG, distearoyl monogalactosyl diglyceride; LNL, α-linolenic acid; 3PH, phosphatidic acid; PAM, palmitoleic acid; SQDG, sulfoquinovosyl diacylglycerol; STE, stearic acid; Chl, chlorophyll; Pheo, pheophytin.

Subunit	Chlorophylls	Carotenoids	Lipids	Others
**PsbA**	4 chl a	1 β-carotene	4 LHG	1 $CaMn_4O_5$
	1 pheo		1 LMG	
			1 LNL	
			1 SQDG	1 plastoquinone
**PsbB**	16 chl a	3 β-carotenes		
			2 LMG	
			1 3PH	
			1 STE	
**PsbC**	13 chl a	3 β-carotenes	3 DGDG	1 Cl^- ion
			1 LHG	
			2 LMG	
			2 LNL	
**PsbD**	2 chl a	1 β-carotene	3 LHG	1 bicarbonate ion
	1 pheo		2 LMG	1 plastoquinone
				1 Fe^{3+} ion
			2 SQDG	
**PsbE**				
**PsbF**				1 heme
**PsbH**		1 β-carotene	1 LMG	
			1 LNL	
**PsbI**			1 LNL	
**PsbJ**		1 β-carotene	1 LMG	
**PsbK**			1 LMG	
**PsbL**			1 LHG	
			1 SQD	
**PsbM**				
**PsbO**				
**PsbP**				
**PsbQ**				

**PsbR**			1 3PH	
**PsbT**			1 3PH	
**Ycf12 (Psb30)**				
**PsbW**			1 DGDG<br>2 LNL	
**PsbX**			1 LNL	
**PsbY**				
**PsbZ**				
**Lhcb4**	11 chl a<br>3 chl b	1 Neoxanthin<br>1 Lutein<br>1 Violaxanthin	3 LHG<br>1 LNL	
**Lhcb5**	11 chl a<br>4 chl b	1 Neoxanthin<br>2 Luteins	2 LHG<br>1 LMG	
**S-LHCII**	26 chl a<br>18 chl b	3 Neoxanthins<br>6 Luteins<br>1 Violaxanthin	5 LHG<br>2 LNL<br>1 PAM	
**M-LHCII**	24 chl a<br>18 chl b	3 Neoxanthins<br>6 Luteins<br>1 Violaxanthin	3 LHG	
**L-LHCII**	25 chl a<br>17 chl b	3 Neoxanthins<br>6 Luteins<br>1 Violaxanthin	2 LHG	
**Total**	192 chls, 2 pheo	46	59	7

Supplementary Table 3 | Interactions of novel subunits PsbR and PsbY with the subunits of PSII core complex in *C. ohadii*. Interface area and amino acid residues involved in the specific bonds (hydrogen bond and salt bridges) are indicated.

Subunit interactions	Interface area [Å^2]	H-bonds	Salt bridges
**PsbR-PsbC**	682	Asn51-Glu29	Arg70-Glu456
		Lys57-Glu29	Lys73-Asp460
		Thr58-Glu29	Arg79-Asp462
		Trp64-Ser30	
		Gln68-Arg447	
		Gln68-Glu456	
		Arg70-Glu456	
		Lys73-Asp460	
		Arg79-Asp462	
**PsbR-PsbD**	737	Gly76-Asp227	Lys73-Glu224
		Asn51-Arg233	
		Lys73-Glu224	
		Lys73-Asp227	
		Gly74-Asp227	
		Tyr75-Asp227	
		Gly76-Asp227	
		Tyr78-Asn236	
		Tyr78-Gln239	
		Arg79-Asp225	
		Tyr84-Ala240	
		Tyr84-Glu241	
		Ala86-Asn236	
		Asn87-Phe235	
		Asn87-Asn236	
		Asn87-Arg233	
		Val88-Phe235	
**PsbR-PsbE**	707	Ser101-Asp12	
		Ser103-Arg8	

		Thr135-Ser39	
**PsbR-PsbF**	170	Thr99-Tyr9<br>Ser101-Tyr7<br>Glu102-Ser6	
**PsbR-PsbJ**	289	Asp105-Gly8<br>Tyr107-Thr6	
**PsbR-PsbK**	105	Asn51-Arg42	
**PsbR-PsbP**	147	Ala137-Val480	
**PsbR-PsbY**	298	Tyr107-Asn342	
**PsbY-PsbE**	527	Arg324-Asp45<br>Arg324-Thr40<br>Trp338-Val21	Arg324-Asp45
**PsbY-PsbF**	181	Gln350-Phe12	

Supplementary Table 4 | Binding interactions between light-harvesting proteins and subunits of the photosystem II core complex from *C. ohadii*. Interface area and specific bonds (hydrogen bonds and salt bridges) between amino-acids are indicated. Subscripts represent the label of the specific chain in the pdb file.

Subunit interactions	Interface area [Å^2]	H bonds	Salt bridge
**S-LHCII$_y$ - CP43**	105	Glu241-Ser205	
**S-LHCII$_y$ - PsbW**	130	Tyr110-Trp411	
		Lys205-Tyr425	
**S-LHCII$_n$ - Lhcb5**	772	Tyr154-Trp43	Glu172-Arg39
		Asn157-Ser46	
		Glu163-Ser38	
		Glu172-Arg39	
		Pro176-Gly199	
**S-LHCII$_y$ - Lhcb5**	110	Pro48-Ser46	
		Asp51-Ser46	
**M-LHCII$_3$ - Lhcb4**	517	Tyr162-Gly67	Asp189-Arg253
		Asp183-Arg253	
		Pro184-Arg253	
**L-LHCII$_{13}$ - Lhcb4**	630	Asn128-Phe293	Asp181-Arg69
		Asp175-Arg69	Glu172-Arg69
		Asp181-Arg69	
		Glu172-Arg69	
		Glu172-Tyr71	
		Pro160-Asn80	
**Lhcb4-CP47**	844	Asp112-Arg127	Asp112-Arg127
		Gln118-Thr10	
		Tyr193-Leu161	
**Lhcb4-PsbH**	489	Leu109-Val34	
		Asp114-Tyr31	
		Ile111-Gly32	
**Lhcb4-D1**	240	Gln118-Asn230	
		Asn119-Asn230	
		Ala120-Glu229	
**Lhcb4-PsbL**	66	Gln118-Gln9	
		Asn119-Gln9	
**Lhcb5-CP43**	414	Val292-Thr108	Arg289-Asp107
		Arg289-Asp107	
		Val292-Asp107	
**Lhcb5-PsbZ**	342	Leu281-Ser59	

Supplementary Table 5 | Primers used for cloning.

Name	Sequence
Chain O_FW	5´- ATGCTCGCTGCCACTGCCC – 3´
Chain O_REV	5´- CAGCTGAGCTGGCCGTACC – 3´
Chain S_FW1	5´- ATGGTCGCCATGGGCTGCTC – 3´
Chain S_FW2	5´- CCAAGAAGGCCGCAAGTC – 3´
Chain S_REV	5´- TTAGAGCACAGCGGCGCGG – 3´
Chain G_FW	5´- GCCCGCAAGCAGGTGACCCG – 3´
Chain G_REV	5´- TCAGGGCACGCGCAGGAAC – 3´

Supplementary Table 6 | Calculated FRET rates for the excitation energy transfer from individual subunits of LHCII to the core complex of photosystem II from *C. ohadii*. The lifetime (τ) is defined as $1/k_{FRET}$. Designation of the chain in bracket refers to the assignment of the chain in the PDB file 9HD7.pdb. Source data are provided as a Source Data file.

Interacting subunits	C. ohadii	
	FRET rate k_{FRET} (ps^{-1})	lifetime τ (ps)
L-LHCII (chain 16) → Lhcb4 (chain r)	0.030	34
L-LHCII (chain 14) → Lhcb4 (chain r)	0.001	675
L-LHCII (chain 16) → M-LHCII (chain 6)	0.001	860
L-LHCII (chain 16) → M-LHCII (chain 4)	0.098	10
M-LHCII (chain 6) → Lhcb4 (chain r)	0.039	26
M-LHCII (chain 6) → S-LHCII (chain G)	0.249	4
M-LHCII (chain 6) → S-LHCII (chain Y)	0.001	1274
M-LHCII (chain 5) → S-LHCII (chain G)	0.001	789
M-LHCII (chain 6) → L-LHCII (chain 16)	0.001	864
M-LHCII (chain 4) → L-LHCII (chain 16)	0.110	9
S-LHCII (chain N) → Lhcb5 (chain S)	0.021	47
S-LHCII (chain Y) → CP43 (chain C)	0.073	14
S-LHCII (chain Y) → Lhcb4 (chain r)	0.002	498
S-LHCII (chain Y) → M-LHCII (chain 6)	0.001	1276
S-LHCII (chain G) → M-LHCII (chain 5)	0.001	890
S-LHCII (chain G) → M-LHCII (chain 6)	0.278	4
Lhcb5 (chain S) → S-LHCII (chain N)	0.018	56
Lhcb5 (chain S) → CP43 (chain C)	0.085	12
Lhcb4 (chain r) → CP47 (chain b)	0.258	4
Lhcb4 (chain r) → S-LHCII (chain Y)	0.002	583
Lhcb4 (chain r) → M-LHCII (chain 6)	0.033	30
Lhcb4 (chain r) → L-LHCII (chain 16)	0.026	39
Lhcb4 (chain r) → L-LHCII (chain 14)	0.001	880

Supplementary Table 7 | LC-MS/MS analysis of polyamines from thylakoid membranes of *C. ohadii* grown under 100 µmol photons m^{-2} s^{-1} and 1700 µmol photons m^{-2} s^{-1}. Abbreviations used: Cadaverine (CAD), Putrescine (PUT), Diaminopropane (DAP), Norspermidine (NorSPD), Spermidine (SPD), Homospermidine (HomoSPD), Thermospermine (ThSPM), Spermine (SPM). Source data are provided as a Source Data file.

		analysis #	CAD	PUT	DAP	NorSPD	SPD	HomoSPD	ThSPM	SPM	Sum of all polyamines [fmol/uL]
***C. ohadii* - HL 1700 µmol m^{-2} s^{-1}**	Bounded [fmol/uL]	1	47550	52461	58361	589	28220	377	5	3658	
		2	47094	50497	62332	364	28420	395	7	3891	
		3	45229	48695	59815	475	27396	487	9	5334	
	Free [fmol/uL]	1	110221	96592	83767	3134	54497	476	4	1480	
		2	118542	113435	103018	3888	53694	608	6	1899	
		3	121492	114089	111270	3192	52182	493	4	1409	
	Conjugated [fmol/uL]	1	0	0	0	503	0	0	0	0	
		2	0	0	0	424	0	0	0	0	
		3	0	0	0	489	0	0	0	0	
	Sum of polyamines [fmol/uL]	1	157772	149053	142128	4226	82716	854	9	5138	541896
		2	165636	163932	165350	4675	82114	1003	12	5791	588514
		3	166721	162783	171086	4155	79577	979	13	6743	592058
	**Average of the sum of all polyamines**										**574156**

		analysis #	CAD	PUT	DAP	NorSPD	SPD	HomoSPD	ThSPM	SPM	Sum of all polyamines [fmol/uL]
***C. ohadii* - NL 100 µmol m^{-2} s^{-1}**	Bounded [fmol/uL]	1	247	1702	2424	128	7172	41	3	675	
		2	241	1503	2928	118	8043	62	2	615	
		3	401	1877	2886	88	7812	53	5	827	
	Free [fmol/uL]	1	203	17477	10111	331	429	39	1	135	
		2	231	18170	10916	289	476	28	1	74	
		3	237	17431	10149	259	470	25	1	87	
	Conjugated [fmol/uL]	1	223	0	14218	393	0	7	1	267	
		2	159	0	12606	351	0	9	0	174	
		3	184	0	13217	337	0	10	0	209	
	Sum of polyamines [fmol/uL]	1	673	19178	26753	851	7601	88	4	1077	56227
		2	631	19673	26450	758	8519	99	3	863	56996
		3	821	19309	26252	684	8282	87	6	1123	56565
	**Average of the sum of all polyamines**										**56596**

Supplementary Methods
Characterization of PSII subunits and thylakoid membrane proteins by mass spectrometric analysis

Separated PSII supercomplexes and thylakoid membranes from *C. ohadii* were lysed separately in SDT buffer (4% SDS, 0.1M DTT, 0.1M Tris/HCl, pH 7.6) in a thermomixer (Eppendorf ThermoMixer® C, 15 min, 95°C, 750 rpm). After that, the sample was centrifuged (15 min, 20,000 x g) and the supernatant was divided into 3 replicates (ca 10 µg of total protein each) and used for filter-aided sample preparation (FASP) as described elsewhere[1] using 10 kDA cut-off filter columns and 0.5 µg of trypsin (sequencing grade; Promega). The resulting peptides were analysed by LC-MS/MS.

LC-MS/MS analyses of all peptides were done using the UltiMate 3000 RSLCnano system (Thermo Fisher Scientific) connected to timsTOF Pro spectrometer (Bruker). Prior to LC separation, tryptic digests were online concentrated and desalted using a trapping column (Acclaim™ PepMap™ 100 C18, dimensions 300 µm ID, 5 mm long, 5 µm particles, Thermo Fisher Scientific). After washing the trapping column with 0.1% formic acid (FA), the peptides were eluted (flow rate - 300 nL/min) from the trapping column onto an analytical column (Aurora C18, 75µm ID, 250 mm long, 1.6 µm particles, heated to 50°C, Ion Opticks) by 90 min linear gradient program (3-42% of mobile phase B; mobile phase A: 0.1% FA in water; mobile phase B: 0.1% FA in 80% ACN). Equilibration of the trapping column and the analytical column was done prior to sample injection into the sample loop. The analytical column was placed inside the Butterfly Heater (Phoenix S&T) and its emitter side was installed inside the CaptiveSpray ion source (Bruker) according to the manufacturer's instructions with the column temperature set to 50 °C.

MSn data were acquired in data-independent acquisition (DIA) mode with base method m/z range of 100-1700 and 1/k0 range of 0.6-1.6 V×s×cm^{-2}. The precursor range was defined to m/z 400-1000 with equal windows sizes of 26 Th using two steps each PASEF scan and cycle time of 100ms locked to 100% duty cycle.

DiaPASEF data were processed in DIA-NN[2] (version 1.8) in library-free mode against the modified cRAP database (based on http://www.thegpm.org/crap; 111 sequences; prepared 11-2018), *C. ohadii* database (based on https://www.ncbi.nlm.nih.gov/ipg/?term=txid2649997[Organism:noexp]; 11,404 sequences, prepared 04-2023) and custom protein database for *C. ohadii* PSII supercomplex (based on a cryo EM model deposited under the accession number 9HD7.pdb, 29 protein sequences, prepared 10-2023). No variable, carbamidomethylation as fixed modification and trypsin/P enzyme with 1 allowed missed cleavages were set during the library preparation. False discovery rate (FDR) control was set to 1% FDR. MS1 and MS2 accuracies as well as scan window parameters were set based on the initial test searches (median value from all samples ascertained parameter values). MBR was switched on.

Protein MaxLFQ intensities reported in the DIA-NN main report file were further processed using the software container environment (https://github.com/OmicsWorkflows), version 4.6.3a. The processing workflow is available upon request. Briefly, it covered: a) removal of low-quality precursors and contaminant protein groups, b) protein group intensities log2 transformation and normalization.

Mass spectrometry proteomics data were deposited to the ProteomeXchange Consortium via PRIDE[3] partner repository under dataset identifier PXD059509.

Supplementary References

1. Wisniewski, R., Zougman, A., Nagaraj, N., Mann, M. Universal sample preparation method for proteome analysis. *Nat. Methods* **5**, 359-362 (2009).
2. Demichev, V., *et al*. Neural Networks and Interference Correction Enable Deep Proteome Coverage in High Throughput. *Nat. Methods* **17**, 41–44 (2020).
3. Perez-Riverol, Y., Csordas, A., Bai, J., Bernal-Llinares, M., Hewapathirana, S., Kundu, D.J., *et al.* The PRIDE database and related tools and resources in 2019: improving support for quantification data. *Nucleic Acids Res.* **47,** D442–D450 (2019).
