# Supplementary material for: Cryo-EM structure of photosystem II supercomplex from a green microalga with extreme phototolerance
Source: Nat Commun. 2026 Jan 9;17:341. doi: 10.1038/s41467-025-65861-2 (PMC12789144; doi:10.1038/s41467-025-65861-2)
Supplement: Supplementary file 3 — Reporting Summary [file 41467_2025_65861_MOESM3_ESM.pdf]

nature portfolio

Corresponding author(s): Roman Kouřil

Last updated by author(s): Jul 9, 2025

Reporting Summary

Nature Portfolio wishes to improve the reproducibility of the work that we publish. This form provides structure for consistency and transparency in reporting. For further information on Nature Portfolio policies, see our Editorial Policies and the Editorial Policy Checklist.

Statistics

For all statistical analyses, confirm that the following items are present in the figure legend, table legend, main text, or Methods section.

n/a	Confirmed	
☐	☒	The exact sample size (*n*) for each experimental group/condition, given as a discrete number and unit of measurement
☐	☒	A statement on whether measurements were taken from distinct samples or whether the same sample was measured repeatedly
☒	☐	The statistical test(s) used AND whether they are one- or two-sided<br>*Only common tests should be described solely by name; describe more complex techniques in the Methods section.*
☒	☐	A description of all covariates tested
☒	☐	A description of any assumptions or corrections, such as tests of normality and adjustment for multiple comparisons
☒	☐	A full description of the statistical parameters including central tendency (e.g. means) or other basic estimates (e.g. regression coefficient) AND variation (e.g. standard deviation) or associated estimates of uncertainty (e.g. confidence intervals)
☒	☐	For null hypothesis testing, the test statistic (e.g. *F*, *t*, *r*) with confidence intervals, effect sizes, degrees of freedom and *P* value noted<br>*Give P values as exact values whenever suitable.*
☒	☐	For Bayesian analysis, information on the choice of priors and Markov chain Monte Carlo settings
☒	☐	For hierarchical and complex designs, identification of the appropriate level for tests and full reporting of outcomes
☒	☐	Estimates of effect sizes (e.g. Cohen's *d*, Pearson's *r*), indicating how they were calculated

Our web collection on statistics for biologists contains articles on many of the points above.

Software and code

Policy information about availability of computer code

Data collection	The acquisition of electron micrographs was performed using the EPU3.3 software and the cryoSPARC Live software was employed for on-the-fly analysis. For acquisition of LC-MS/MS data (proteomic) Bruker Compass HyStar 6.2 software was used.
Data analysis	Single particle cryo-EM analysis was performed using cryoSPARC v4.3.1. Initial fitting of the subunits in the cryo-EM map was performed by rigid body real-space refinement in ChimeraX 1.9. Local fitting of the subunits in the cryo-EM map was performed using the program Coot 0.9.8. and refinement was performed in Phenix 1.21.2_5419. The validation statistics were calculated by MolProbity 4.5.2. Images were prepared with PyMOL 2.5.2. The interface area between specific protein subunits and between cofactors was calculated from structural files using the Pisa software. FRET analysis was performed using MS-Excel. Global analysis of time-resolved spectroscopy data was performed using the pyglotaran python package.

For manuscripts utilizing custom algorithms or software that are central to the research but not yet described in published literature, software must be made available to editors and reviewers. We strongly encourage code deposition in a community repository (e.g. GitHub). See the Nature Portfolio guidelines for submitting code & software for further information.

Data

Policy information about availability of data

All manuscripts must include a data availability statement. This statement should provide the following information, where applicable:

- Accession codes, unique identifiers, or web links for publicly available datasets
- A description of any restrictions on data availability
- For clinical datasets or third party data, please ensure that the statement adheres to our policy

The cryo-EM map of C. ohadii PSII-LHCII supercomplex has been deposited in the Electron Microscopy Data Bank with accession codes EMD-52056.
The structure model of C2S2M2L2 supercomplex is deposited in the PDB under the accession code 9HD7.
DNA sequences coding for chains G/g, O/o, and S/s were deposited to GenBank with accession codes PQ455547, PQ455546 and PQ456901.
Mass spectrometry proteomics data were deposited to the ProteomeXchange Consortium via PRIDE partner repository under dataset identifier PXD059509.

Research involving human participants, their data, or biological material

Policy information about studies with human participants or human data. See also policy information about sex, gender (identity/presentation), and sexual orientation and race, ethnicity and racism.

Reporting on sex and gender	N/A.
Reporting on race, ethnicity, or other socially relevant groupings	N/A.
Population characteristics	N/A.
Recruitment	N/A.
Ethics oversight	N/A.

Note that full information on the approval of the study protocol must also be provided in the manuscript.

Field-specific reporting

Please select the one below that is the best fit for your research. If you are not sure, read the appropriate sections before making your selection.

☒ Life sciences ☐ Behavioural & social sciences ☐ Ecological, evolutionary & environmental sciences

For a reference copy of the document with all sections, see nature.com/documents/nr-reporting-summary-flat.pdf

Life sciences study design

All studies must disclose on these points even when the disclosure is negative.

Sample size	In total, 12,419 movies were recorded and analyzed. No statistical analyses has been performed. The number of cryo-EM particles in the dataset collected was the number of particles available. No predetermined sample size was used for other experiments.
Data exclusions	Particles that were not C2S2M2L2 photosystem II-LHCII supercomplexes were excluded from the dataset during image analysis (2D classification) because they cannot contribute to the reconstruction.
Replication	Isolated photosystem II supercomplexes for cryo-EM analysis were obtained from more than 12 sucrose gradient tubes. Preliminary EM analysis of negatively stained particles confirmed the presence of photosystem II supercomplexes in the isolated sucrose gradient fraction. Six grids were prepared and screened for cryo-EM analysis. The best cryo specimen was used for the data acquisition. The cryo-EM data collection have not been repeated. LC-MS/MS measurement (proteomic) was done in technical triplicates. Estimation of polyamines by LC-MS/MS analysis was done in technical triplicates. Biological variability was achieved when multiple cell cultures were harvested.
Randomization	We utilized the Gold standard Fourier Shell Correlation method to assess the resolution of the cryo-EM structures. This method involves splitting the dataset into two sets, odd and even, which are then refined independently. The splitting of the dataset is random.
Blinding	N/A to cryo-EM study, therefore no blinding was done in this study. All models were generated ab-initio with the maximal number of available cryo-EM particles.

Reporting for specific materials, systems and methods

We require information from authors about some types of materials, experimental systems and methods used in many studies. Here, indicate whether each material, system or method listed is relevant to your study. If you are not sure if a list item applies to your research, read the appropriate section before selecting a response.

Materials & experimental systems

n/a	Involved in the study
☒	☐ Antibodies
☒	☐ Eukaryotic cell lines
☒	☐ Palaeontology and archaeology
☒	☐ Animals and other organisms
☒	☐ Clinical data
☒	☐ Dual use research of concern
☐	☒ Plants

Methods

n/a	Involved in the study
☒	☐ ChIP-seq
☒	☐ Flow cytometry
☒	☐ MRI-based neuroimaging

Plants

Seed stocks	A cell culture of Chlorella ohadii was provided by Prof. Aaron Kaplan, the Hebrew University of Jerusalem, Israel.
Novel plant genotypes	N/A.
Authentication	N/A.
